# Nivolumab Reduces PD1 Expression and Alters Density and Proliferation of Tumor Infiltrating Immune Cells in a Tissue Slice Culture Model of Renal Cell Carcinoma

**DOI:** 10.3390/cancers13184511

**Published:** 2021-09-08

**Authors:** Philipp J. Stenzel, Nina Hörner, Sebastian Foersch, Daniel-Christoph Wagner, Igor Tsaur, Anita Thomas, Axel Haferkamp, Stephan Macher-Goeppinger, Wilfried Roth, Stefan Porubsky, Katrin E. Tagscherer

**Affiliations:** 1 Institute of Pathology, University Medical Center Mainz, 55131 Mainz, Germany; nina.hoerner@unimedizin-mainz.de (N.H.); sebastian.foersch@unimedizin-mainz.de (S.F.); Daniel-Christoph.Wagner@unimedizin-mainz.de (D.-C.W.); sgoeppinger@gmail.com (S.M.-G.); Wilfried.Roth@unimedizin-mainz.de (W.R.); stefan.porubsky@unimedizin-mainz.de (S.P.); katrin.tagscherer@unimedizin-mainz.de (K.E.T.); 2 Department of Urology, University Medical Center Mainz, 55131 Mainz, Germany; igor.tsaur@unimedizin-mainz.de (I.T.); anita.thomas@unimedizin-mainz.de (A.T.); axel.haferkamp@unimedizin-mainz.de (A.H.)

**Keywords:** nivolumab, clear cell renal cell carcinoma, tissue slice culture, PD1, T-cells

## Abstract

**Simple Summary:**

Immune checkpoint inhibitors (ICIs) have become a first-choice therapy option in the treatment of clear cell renal cell carcinoma (ccRCC). A predictive biomarker is urgently needed since not all patients respond and adverse events occur. Therefore, an ex vivo tissue slice culture (TSC) model was tested to investigate the effects of nivolumab on tumor infiltrating immune cells (TIIC). A decrease in programmed death receptor 1 expression, as well as effects on density and proliferation of TIIC, were observed. Thus, the TSC model could serve as a test platform for response prediction to ICIs.

**Abstract:**

Background: In the treatment of clear cell renal cell carcinoma (ccRCC), nivolumab is an established component of the first-line therapy with a favorable impact on progression free survival and overall survival. However, treatment-related adverse effects occur and, to date, there is no approved predictive biomarker for patient stratification. Thus, the aim of this study was to establish an ex vivo tissue slice culture model of ccRCC and to elucidate the impact of nivolumab on tumor infiltrating immune cells. Methods: Fresh tumor tissue of ccRCC was treated with the immune checkpoint inhibitor nivolumab using ex vivo tissue slice culture (TSC). After cultivation, tissue slices were formalin-fixed, immunohistochemically stained and analyzed via digital image analysis. Results: The TSC model was shown to be suitable for ex vivo pharmacological experiments on intratumoral immune cells in ccRCC. PD1 expression on tumor infiltrating immune cells was dose-dependently reduced after nivolumab treatment (*p* < 0.01), whereas density and proliferation of tumor infiltrating T-cells and cytotoxic T-cells were inter-individually altered with a remarkable variability. Tumor cell proliferation was not affected by nivolumab. Conclusions: This study could demonstrate nivolumab-dependent effects on PD1 expression and tumor infiltrating T-cells in TSC of ccRCC. This is in line with results from other scientific studies about changes in immune cell proliferation in peripheral blood in response to nivolumab. Thus, TSC of ccRCC could be a further step to personalized medicine in terms of testing the response of individual patients to nivolumab.

## 1. Introduction

Renal cell carcinoma (RCC) is among the ten most frequent malignancies worldwide with increasing incidence and decreasing mortality [1]. The decrease in mortality is the consequence of early diagnosis and a broad range of therapy options in advanced and metastasized stages, which applies to 20% of patients at the time of diagnosis and another 20% of patients during the clinical course after initial surgery [2]. For clear cell renal cell carcinoma (ccRCC), the most common histologic subtype of RCC, the combination of either tyrosine kinase inhibitor (TKI) and immune checkpoint inhibitor (ICI) or two ICIs are guideline-recommended first-line treatment options. Four comprehensive clinical trials (Checkmate 214 (ClinicalTrials.gov Identifier: NCT02231749), Keynote 426 (ClinicalTrials.gov Identifier: NCT02853331), Javelin 101 (ClinicalTrials.gov Identifier: NCT02684006), Checkmate9ER (ClinicalTrials.gov Identifier: NCT03141177)) have shown the superiority of either combined ICI therapy (nivolumab + ipilimumab) or combined TKI and ICI therapy (axitinib + pembrolizumab, axitinib + avelumab, cabozantinib + nivolumab) regarding overall survival (OS) or progression free survival (PFS) compared to standard-of-care sunitinib in patients with previously untreated advanced RCC [3,4,5,6]. The objective response rate (ORR) for the combination therapies including ICIs ranged from 42% [3] to 59.3% [4] compared to 25.7% to 35.7% for sunitinib alone [3,4,5,6]. Complete responses were rare in all studies. Treatment-related adverse events of grade 3 or higher occurred either in the sunitinib group [3] or in the combination therapy group [4,6] or showed no significant difference [5]. With the exception of the Javelin 101 trial, treatment was discontinued due to treatment-related adverse effects, more often in the group with combination therapy. In the Checkmate 214 trial, there were even eight treatment-related deaths in the group treated with the combination of ipilimumab and nivolumab compared to four treatment-related deaths in the sunitinib group [3].

Hence, stratifying patients eligible for therapy including ICIs remains a difficult task and predictive biomarkers are urgently needed. Programmed death receptor ligand 1 (PD-L1) expression of RCC has been examined in the above mentioned clinical trials, but has not been established as a reliable predictive biomarker for ICI [7]. A more dynamic approach is to measure blood parameters before or during ICI treatment. Serum levels of soluble programmed death receptor 1 (sPD1) and sPD-L1 correlated with OS and PFS of patients with RCC [8,9]. In patients with non-small cell lung cancer (NSCLC) an increased proliferation of CD8+ cytotoxic T-cells (CTL) in peripheral blood correlated with response to nivolumab therapy, whereas patients with progressive disease had no change in CTL proliferation or even a decrease [10,11]. In metastasized RCC, a high density of tumor infiltrating PD1+ CTLs correlated with higher ORR and prolonged PFS in a patient cohort treated with nivolumab and is, therefore, a promising candidate predictive biomarker for response to nivolumab [12,13].

In this study, we report our results regarding an ex vivo tissue slice culture (TSC) model with incubation of fresh vital ccRCC tumor tissue with nivolumab for 24 h or 72 h and consecutive quantification of immune cell density, proliferation, and distribution.

## 2. Materials and Methods

### 2.1. Patients and Tissue Collection

Twelve patients with ccRCC, surgically treated at the Department of Urology and Pediatric Urology of the University Medical Center Mainz from 2017 to 2020, were included in the study. Tissue collection was approved by ethics approval for the Tissue Biobank, University Medical Center Mainz (ethics approval: 837.031.15 (9799); date of approval: 2 October 2015). After arrival of the surgical specimen in the Institute of Pathology, tumors were macroscopically examined to confirm subtype (golden to yellow cut surface with hemorrhage) and tissue vitality. In cases of doubt, additional microscopic examination by frozen section of the intended area of sampling was performed. Exclusion criteria for tissue collection included a tumor size <1.0 cm to ensure reliable pathologic routine diagnostics, poor tissue quality with a high portion of necrotic tumor tissue, and non-clear cell morphology. Fresh vital tumor tissue (length: 10 mm; diameter: 6 mm) was collected from the tumor periphery by using a defined punching tool (KAI Medical Biopsy Punch, Solingen, Germany), stored in a 4 °C chilled Krebs-Henseleit-Buffer (Sigma-Aldrich/Merck, Darmstadt, Germany) and referred to the lab for TSC. To match tumor heterogeneity, at least two different tumor localizations were sampled.

### 2.2. Ex Vivo Tissue Slice Culture

**Figure 1:**
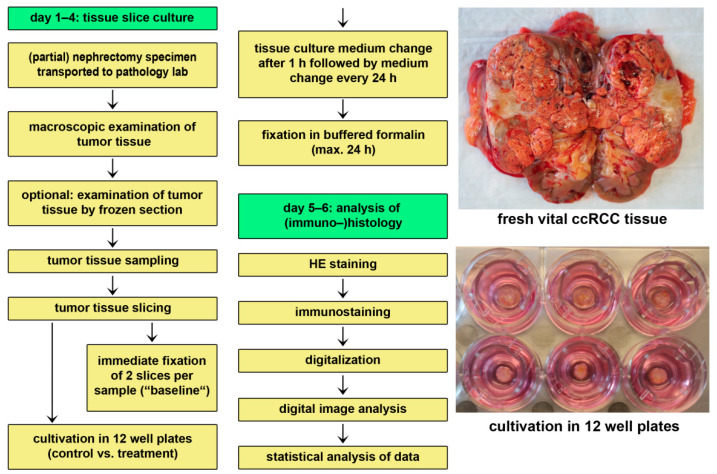
Tissue culture workflow.

The tissue culture protocol has been described in detail previously [14,15]. Briefly, tumor tissue was cut into slices of 300 µm thickness using a Vibratome VT1200 (Leica Microsystems, Mannheim, Germany). The first and the last slice of each tumor sample was immediately fixated in buffered 4% formalin. The other tissue slices were randomly assigned to control and intervention groups. Tissue slices were incubated at the air-medium-interface in a 12-well plate with appropriate inserts. The used tissue culture medium was DMEM cell culture medium (ATCC, Manassas, CO, USA) with supplements (1% Penicillin/Streptomycin, 10% fetal calf serum (Sigma-Aldrich/Merck, Darmstadt, Germany)) and with or without nivolumab (Opdivo, Bristol-Myers Squibb, Munich, Germany). The medium including nivolumab in the therapy group was changed after 1 h and every additional 24 h. For the time of the experiment, tissue slices were kept in an incubator with a humidified atmosphere, a temperature of 37 °C, and 5% CO_2_. After 24 h or 72 h, respectively, tissue slices were harvested, fixated in buffered 4% formalin, and paraffin embedded. Figure 1 provides a detailed overview of the experimental setup.

### 2.3. Treatment Regimen

Tissue slices were incubated with increasing concentrations of nivolumab (0.1 µg/mL, 1 µg/mL, 10 µg/mL, and 100 µg/mL) or without nivolumab as control. Cultivation was usually performed in triplicates and in cases of limited amount of tumor tissue in duplicates.

### 2.4. Conventional and Immunohistochemical Staining

Tissue slices were stained with hematoxilyn and eosin (HE) and tumor tissue vitality was confirmed. Slices with necrosis >50% were excluded from further immunohistochemical stainings. Slices were stained with antibodies against Ki67 (MIB-1, Dako, Glostrup, Denmark), PD-L1 (ab213524, Abcam, Cambridge, UK) or double stained using the Envision G/2 Doublestain System or Envision Flex Doublestain System (Dako). The antibody combinations were CD3 (IR503, Dako) + Ki67 (MIB-1, Dako), CD3 (IR503, Dako) + CK AE1/3 (IR053, Dako), CD8 (IR623, Dako) + Ki67 (MIB-1, Dako), PD1 (ab52587, Abcam) + Ki67 (MIB-1, Dako), and PD1 (ab52587, Abcam) + CK AE1/3 (IR053, Dako). All slides were stained with automatized immunostainers (Autostainer Plus, Dako).

### 2.5. Digital Image Analysis

Digitalization and digital image analysis were performed as previously described using a digital whole slide scanner (Nanozoomer, Hamamatsu Photonics, Hamamatsu, Japan) and the HALO^®^ platform (Indica Labs, Corrales, NM, USA) [16]. Briefly, for the detection and quantification of stain-positive cells, the CytoNuclear module (v1.4–1.6) was used. To differentiate between tumor parenchyma and tumor-associated stroma, a tissue classifier was included. Localization of each detected cell in the tissue and its biomarker profile cells were saved and used for spatial analysis with the proximity tool included in HALO^®^. Vital and necrotic tumor areas were manually annotated and quantified via digital image analysis. PD-L1 status (tumor proportion score, TPS) was assessed by light microscopy (Olympus BX45, Olympus, Tokio, Japan).

### 2.6. Spatial Distribution

Tissue slices were immunohistochemically double stained for T-cells (brown) and for tumor cells (red). Digital image analysis was used for detection of T-cells (markup: red), tumor cells (markup: green), and other cells (markup: blue). Tissue was further classified into tumor area (classifier markup: red) and stroma (classifier markup: green) (Figure S1A). T-cells were dichotomized into “T-cells Tumor” and “T-cells Stroma” depending on the T-cells’ localization (Figure S1B). The percental distribution of T-cells Tumor and T-cells Stroma within a diameter of 30 µm around tumor cells was quantified using the proximity tool implemented in HALO^®^.

### 2.7. Statistical Analysis

Data are given as mean ± standard deviation. In cases of high inter-individual variability of the examined parameters, data were normalized relative to baseline value or to control. For the comparison of two groups, the paired *t*-test was performed and, for the comparison of three or more groups, the one-way analysis of variance (one-way ANOVA) was performed. The necessary assumptions for the one-way ANOVA were tested with the Shapiro–Wilk test (normal distribution within the individual groups) and the Levene test (homogeneity of variances). In cases, where the assumption of normal distribution within the individual groups was violated, the Kruskal–Wallis test was alternatively performed. Post hoc tests for the one-way ANOVA were the Tukey test and, for the Kruskal–Wallis test, the Dunn test. All calculations were performed using Microsoft Excel (version 2012), R statistical software (version 4.0.3) and Rstudio (version 1.4.1103). Differences with *p*-values < 0.05 were considered significant.

## 3. Results

### 3.1. Characteristics of Patient Collective

Tumor tissues from 12 patients were treated with nivolumab for 24 h (tumors 1–7) or 72 h (tumors 8–12), respectively. Eleven specimens were from primary renal tumors and one from an adrenal gland metastasis. 63.6% (*n* = 7) of primary tumors were organ confined. The median age of patients at the moment of surgery was 65 years (mean 68.3 ± 10.0). Clinical follow-up data was available for nine patients. The median time of follow-up was 10.9 months (min. 1 month, max. 22.9 months, and the mean was 11.4 ± 8.3 months). By the end of follow-up, one patient had died of a disease unrelated to RCC, one was suffering from progressive disease, two showed a stable disease, and five showed no progress. Clinicopathological data including follow up are summarized in Table 1.

### 3.2. Tissue Slice Culture Is Possible for up to Three Days but Reduces Tumor Infiltrating Immune Cells

**Figure 2:**
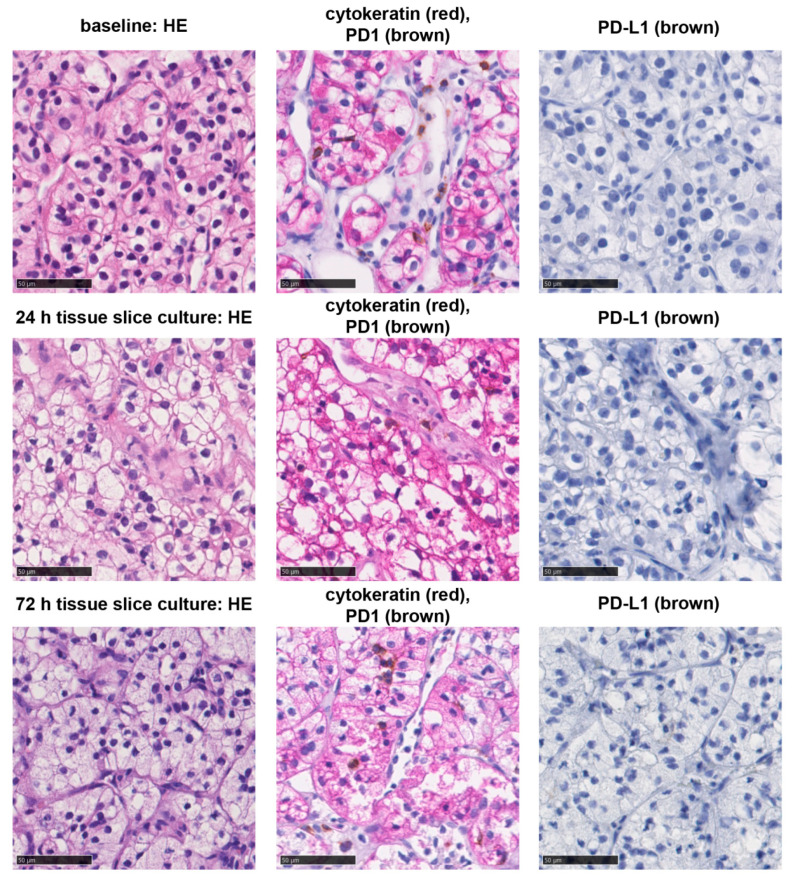
Histological morphology and PD-1/PD-L1 status of tumors. Clear cell morphology of tumors (HE-staining, left), PD1+ immune cells (brown) in the tumor tissue (red) (middle), and PD-L1 expression (brown) at baseline and after tissue slice culture for 24 h or 72 h. Interspersed PD1+ immune cells were present, however the tumor cells showed no PD-L1 expression. Bar indicates 50 µm.

**Figure 3:**
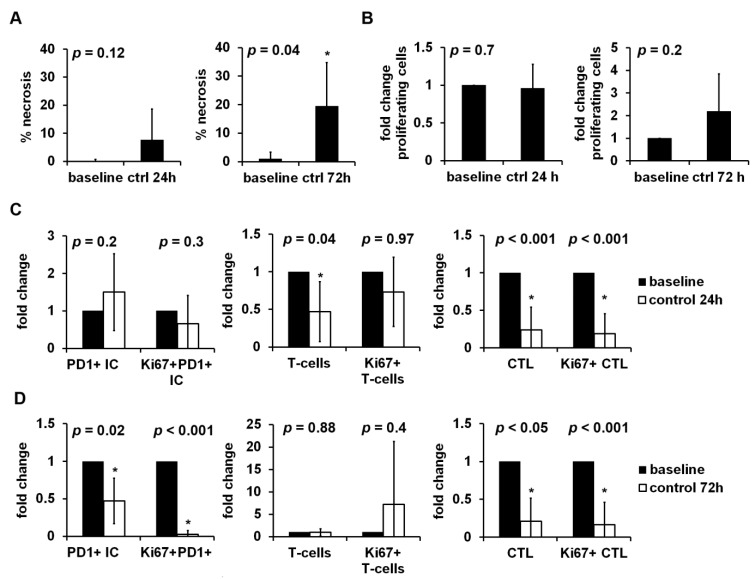
Influence of tissue slice culture (TSC) on tumor vitality, proliferation, and tumor infiltrating immune cells. Tumor tissue of clear cell renal cell carcinoma (ccRCC) was cultivated for 24 h to 72 h. Necrotic tumor area and the immunohistochemical stainings Ki67, Ki67-PD1, Ki67-CD3, and Ki67-CD8 were quantified by digital image analysis. (**A**) The percentage of necrotic tumor after 24 h (*n* = 7) or 72 h (*n* = 5) of TSC compared to baseline. Fold change of (**B**) overall proliferation, (**C**) tumor infiltrating PD1+ immune cells, T-cells, cytotoxic T-cells and their proliferating subsets after 24 h (*n* = 7) or (**D**) 72 h (*n* = 5) of TSC compared to baseline. Data were normalized relative to baseline values, if not stated otherwise, and given as mean ± standard deviation. The paired *t*-test was used for statistical analysis. *: *p* < 0.05.

Fresh vital tumor tissue of ccRCC was sampled close to the invasive margin (IM). Post hoc immunostaining of corresponding primary tumors showed that there were tumors with low (Figure S2A) and high (Figure S2B) amounts of tumor infiltrating PD1+ IC which were rather concentrated at the IM. The tumor tissue was cut into 300 µm thick slices and cultivated with increasing concentrations of nivolumab for 24 h or 72 h, respectively. With only one tumor sample which had to be excluded from further analysis due to extensive cultivation related necrosis, the success rate for the establishment of TSC corresponds to 92.3%. All tumors showed clear cell morphology and stayed negative for PD-L1 during TSC (tumor proportion score: 0%). Tumor infiltrating immune cells with PD1 expression (PD1+ IC) could be detected in every tumor (Figure 2). Baseline densities and proliferation rates of tumor infiltrating immune cells showed a high inter-individual variability (Table 2). After 24 h of TSC, the necrotic tumor area was non-significantly compared to baseline, whereas there was a significant increase in necrosis after 72 h (Figure 3A). Overall proliferation, including tumor cells, showed no significant change after 24 h and a non-significant increase after 72 h (Figure 3B). Tumor infiltrating PD1+ IC, proliferating PD1+ IC and proliferating T-cells were not altered significantly after 24 h of cultivation. Tumor infiltrating T-cells, CTL, and proliferating CTL were significantly decreased (Figure 3C). After 72 h of TSC, PD1+ IC, proliferating PD1+ IC, CTL, and proliferating CTL were all significantly decreased compared to baseline. Tumor infiltrating T-cells were not changed after 72 h of TSC and proliferating T-cells non-significantly increased (Figure 3D).

### 3.3. Distinct Reaction Patterns of Tumor Infiltrating Immune Cells in Response to Nivolumab

**Figure 4:**
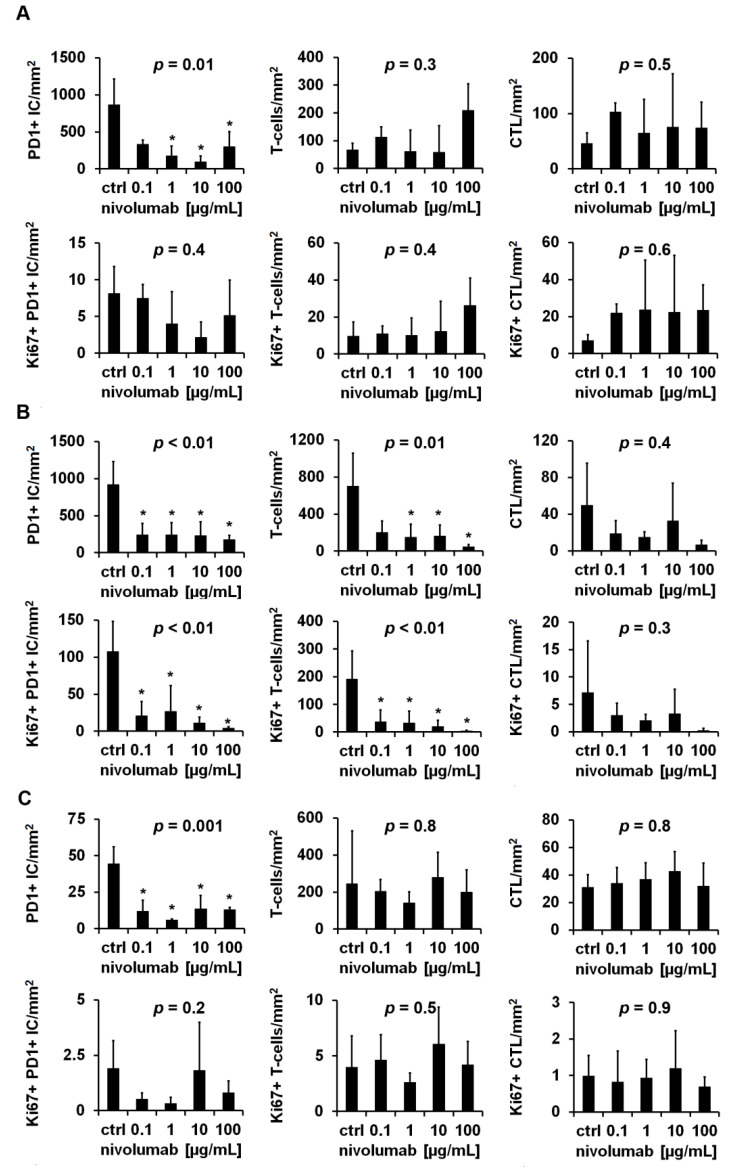
Nivolumab-dependent changes of tumor infiltrating immune cell densities and proliferation rates. Tissue slices of clear cell renal cell carcinoma (ccRCC) were immunohistochemically stained for Ki67-PD1, Ki67-CD3, and Ki67-CD8 after 24 h of cultivation with increasing concentrations of nivolumab. Three representative reaction patterns to nivolumab treatment are shown: (**A**) pattern A (tumor 3) with nivolumab-dependent increased tumor infiltrating T-cells, (**B**) pattern B (tumor 5) with nivolumab-dependent decreased tumor infiltrating T-cells, and (**C**) pattern C (tumor 7) without nivolumab-dependent changes of tumor infiltrating T-cells. Data are given as mean ± standard deviation. For statistical analysis the one-way analysis of variance or the Kruskal–Wallis test with appropriate post hoc tests were used. *p*-values were corrected with the Bonferroni method. *: *p* < 0.05.

A decreased density of PD1+ IC after nivolumab treatment was observed across all examined tumors, whereas T-cells and CTL and the corresponding proliferation fractions showed either a nivolumab dependent increase, decrease, or no alteration. Table 3 provides an overview of the reaction patterns of the individual tumors. Tumor 3 showed a significant decrease in PD1+ IC (*p* = 0.01) and a decrease by trend of proliferating PD1+ IC (*p* = 0.4), a consistent non-significant increase in tumor infiltrating T-cells (*p* = 0.3), proliferating T-cells (*p* = 0.4), CTL (*p* = 0.5), and proliferating CTL (*p* = 0.6) after nivolumab treatment (Figure 4A). In contrast, tumor 5 reacted with a significant decrease in PD1+ IC (*p* < 0.01), proliferating PD1+ IC (*p* < 0.01), T-cells (*p* = 0.01) and proliferating T-cells (*p* < 0.01), as well as a non-significant reduction of CTL (*p* = 0.4) and proliferating CTL (*p* = 0.3) (Figure 4B). Tumor 7 showed the third pattern, characterized by minor, non-significant changes in immune cell densities (T-cells: *p* = 0.8; CTL: *p* = 0.8) and proliferation fractions (Ki67+ PD1+ IC: *p* = 0.2; Ki67 + T-cells: *p* = 0.5; Ki67 + CTL: *p* = 0.9); however, the density of PD1+ IC was significantly decreased (*p* = 0.001) (Figure 4C). Overall proliferation (tumor 3: *p* = 0.99; tumor 5: *p* = 0.1; tumor 7: *p* = 0.3) and nivolumab-dependent necrosis (tumor 3: *p* = 0.1; tumor 5: *p* = 0.7; tumor 7: *p* = 0.5) were not significantly changed (Figure 3). The nivolumab-dependent decrease in PD1+ IC was significant after averaging the respective experiments with a duration of 24 h or 72 h (24 h: *p* < 0.01; 72 h: *p* < 0.01); the other parameters showed no significant changes (Figure 4).

### 3.4. Spatial Distribution of Tumor Infiltrating Immune Cells under the Influence of Nivolumab

**Figure 5:**
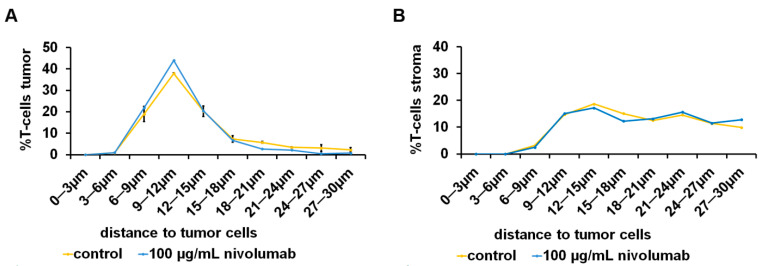
Nivolumab-dependent spatial distribution of tumor infiltrating T-cells. Tumor tissue of clear cell renal cell carcinoma (ccRCC) cultivated 72 h with nivolumab. Tissue slices were immunohistochemically double stained for cytokeratin and CD3. The distance between (**A**) tumor infiltrating T-cells and (**B**) stromal T-cells to tumor cells was calculated by digital image analysis. Data are given as mean ± standard deviation.

In individual experiments, e.g., tumor 11, a minor shifting of tumor infiltrating T-cells toward tumor cells after treatment with 100 µg/mL nivolumab could be observed (Figure 5A), whereas there was a change in the distribution of stromal T-cells (Figure 5B). After averaging the experiments with a duration of 24 h, the distribution of tumor infiltrating PD1+ IC after nivolumab treatment was unaltered compared to control and the T-cells showed a non-significant shift toward tumor cells (Figure S5A). This effect was even more pronounced, yet still not significant, when looking at the corresponding stromal T-cell fraction (Figure S5A), whereas the stromal PD1+ IC were farther away compared to control. After 72 h, there was no major difference in tumor infiltrating T-cells after nivolumab treatment compared to control (Figure S5B).

## 4. Discussion

So far, there are no established predictive biomarkers to guide treatment in metastatic RCC. Therefore, a precise test system for response prediction is one option to better stratify patients who will benefit from nivolumab treatment. In this study, an ex vivo TSC model was tested to examine nivolumab-dependent effects on tumor infiltrating immune cells in human ccRCC tumor tissue.

The major finding of this study was the nivolumab-dependent significant reduction of PD1+ IC (Figure 4 and Figure S3). Immunohistochemical PD1 positivity of tumor infiltrating immune cells is widely considered as a biomarker for “exhausted immune cells”, but with regard to T-cells and especially CTL, it is rather a biomarker for activated CTL [17,18]. In our previously published study about the prognostic value of tumor infitrating immune cells in ccRCC, PD1+ IC were associated with a favorable cancer-specific survival [16], indicative that PD1+ CTL comprise tumor-reactive CTL, as was shown in malignant melanoma [19]. Reduced PD1 expression after incubation with PD1-targeting agents, as described in the present study, has been demonstrated before: PD1 expression of peripheral CD8+ T-Cells of patients suffering from PDAC was reduced after incubation with nivolumab and also with pembrolizumab, another therapeutic anti-PD1-antibody [20]. In a xenograft mouse model of colon carcinoma and mammary carcinoma, a reduced frequency of PD1 + CD8+ T-cells and a decrease in PD1 levels below a certain threshold were associated with release from adaptive immune resistance [21]. In principle, this mechanism could also apply to ccRCC TSC. However, methods other than immunohistochemistry (IHC) are required for a more precise PD1 quantification, e.g., flow cytometry. So far, the decreased density in PD1+ IC after nivolumab treatment, assessed by IHC, could serve as a positive control for a successful nivolumab treatment in TSC. For further interpretation of nivolumab-dependent effects, the focus of this study was on the differentially altered densities and proliferation rates of T-Cells and CTL after nivolumab treatment (Figure 4). This is in line with results examining proliferation of peripheral PD1+ CTL in response to nivolumab in NSCLC. Reduced CTL proliferation after nivolumab infusion correlated with progressive disease [10] and an early proliferative response of PD1+ CTL with (partial) response [11,22]. All in all, the number of tumors (*n* = 12) examined in this study was too small for further correlation of the observed reaction patterns and the clinical course of the included patients. Thus, for further validation of the reported results, these experiments need to be conducted in a larger patient cohort.

All of the examined tumors were immunohistochemically negative for PD-L1. However, PD-L1 expression in renal cell carcinoma has been shown to be a strong prognostic factor for poor outcome [23], but it only provides limited value on response prediction to nivolumab. The Checkmate025 [NCT01668784] trial showed the superiority of nivolumab over everolimus as a second-line therapy for patients with advanced RCC independent of PD-L1 expression [24]. Similarly, the consecutive Checkmate214 and Checkmate9ER trials showed the greater benefit of patients with RCC treated with combination therapies including nivolumab compared to the standard of care sunitinib, independent of the PD-L1 status of the primary tumors [3,6]. Thus, a lack of PD-L1 in the tumor tissue seems to have no major impact on response to nivolumab. Therefore, it is reasonable to investigate the effect of nivolumab on tumor infiltrating immune cells in PD-L1 negative ccRCC tumors, too.

The used ex vivo TSC bears several advantages to address this question compared to other established lab-based experimental designs. Firstly, primary and metastatic tumor tissue can be examined with TSC. Cultivation of several samples from different localizations within the tumor tissue allows for modelling tumor heterogeneity, especially with regard to PD-L1 expression [25]. Thus, at least two samples from different tumor localizations were taken. On the other hand, high tumor heterogeneity can result in the high variance of measured data; despite the lack of significant tendencies due to the high variances, these results should also be interpreted as representative for the whole tumor, because they comprise several tumor localizations. Secondly, the tumor microenvironment (TME) which is crucial for interactions between tumor cells and tumor-associated immune cells is transferred into TSC so that associations between therapy effects, e.g., necrosis or proliferation of tumor cells, and the TME can be discovered. One study using the TSC method for pharmacological experiments on RCC highlights the importance of PD-L1 expression and tumor infiltrating CTL [26]. Investigation in the TME can indeed be achieved with air-liquid interface patient-derived organoids or humanized mouse models, too, but establishing these is resource and time intensive [27,28]. Third, differently to common in vitro monolayer cell culture models, the three-dimensional architecture of the tumor is preserved in TSC. This leads to the conception that the tumor tissue reacts similarly in an ex vivo setting compared to the in vivo situation [29,30,31]. The TSC protocol used in this study has been established for ccRCC tumor tissue and was successfully used in a previous study in our lab (14). Nonetheless, there was increased necrosis of tumor tissue, unrelated to nivolumab treatment (Figure 3A). Tumor necrosis is not uncommon in ccRCC and is a poor prognostic marker for survival [32]. Therefore, a certain amount of tissue necrosis in TSC must be considered inevitable when screened for drug response and can, in principle, be kept low with experiment durations of 24 h (Figure 3A). In terms of the optimization of TSC, protocols studies have so far focused on improving TSC media compositions with regard to tumor cell vitality [33]. Overall proliferation was not significantly changed (Figure 3B) which implies that the used TSC medium composition is suitable to maintain tumor cell proliferation. In contrast, there was a marked drop of immune cell density and proliferation due to TSC alone (Figure 3C,D). CTL were extraordinarily affected which could explain the lack of nivolumab-dependent tumor necrosis in this study (Figure S3). While most TSC studies focus on effects on tumor cells, two have examined tumor infiltrating immune cells in ductal pancreatic adenocarcinoma and gastric carcinoma and found no significant reduction up to day 6 of TSC [34,35]. Thus, in further projects, TSC medium composition should be reevaluated to support both tumor cell and immune cell vitality and proliferation.

In malignant melanoma, responders to ICI had significantly higher CTL densities within 20 µm around tumor cells compared to non-responders [36]. In this study, the spatial distribution of immune cells regarding the distance to tumor cells was investigated, too. The data show only minor nivolumab-dependent effects on the distribution of tumor infiltrating T-cells and PD1+ IC within 30 µm around tumor cells (Figure 5 and Figure S5). This is most likely due to the fact that the ccRCC tumor tissue punches that are used for TSC lack abundant tumor-associated stroma, which means that tumor infiltrating immune cells are close to tumor cells at any time during the experiment. Additionally, intact tissue slices are necessary for the measurement of spatial distribution since tearing of tissue slices is a major confounder. To circumvent these issues, live cell imaging of CTL, as was already established for lung tumor TSC [37], could provide a more detailed insight into the influence of nivolumab on CTL migration through the tumor and number of contacts to tumor cells.

The limitations of the study are: 1. The relatively low number of cases which are sufficient to document the potential and pitfalls of this method but is too low to prove that response to nivolumab can be predicted through TSC and the measurement of tumor infiltrating immune cells; 2. The implementation of clinical studies to correlate the results of this model with clinical therapies and outcomes is needed; 3. The tumor-inherent heterogeneity that—as discussed above—makes sampling at different locations necessary to achieve reliable results. This can turn out to be problematic especially in cases with poor tissue quality or large necrosis.

## 5. Conclusions

Taken together, the present study provides encouraging data that support the ex vivo TSC approach as a model to predict response to nivolumab in ccRCC. Yet, TSC conditions must be optimized in order to minimize effects on tumor infiltrating immune cells through TSC alone. This together with further research on the correlation of nivolumab-dependent changes in immune cell proliferation as a readout parameter for response of ccRCC patients to nivolumab treatment might be the way to establish TSC as a predictive test system.

## Figures and Tables

**Table 1:** Clinicopathological information of the patient collective.

Tumor Number	Sex	Age at Surgery	Origin	Tumor Size (cm)	TNM Classification	Grading	Clinical Course after Surgery
1	m	61	Primary tumor	12	pT2b, pNX, cM0, L0, V0, Pn0, R0	G2	Deceased (unrelated to RCC)
2	m	61	Primary tumor	5.5	pT1b, pNX, cM0, L0, V0, Pn0, R0	G1	No information
3	m	82	Primary tumor	3.4	pT3a, pNX, cM0, L0, V0, Pn0, R0	G2	No progress
4	m	64	Primary tumor	3.7	pT1a, pNx, cM0, L0, V0, Pn0, R0	G2	No information
5	m	60	Metastasis	1.2	pT3a, pN0 (0/1), pM1 (ADR), L0, V1, Pn0, R0	G2	Stable disease
6	m	66	Primary tumor	5	pT1b, pNx, cM0, L0, V0, Pn0, R0	G2	No progress
7	m	72	Primary tumor	8	pT3b, pN0(0/1), cM0, L0, V2, Pn0, R1	G2	No progress
8	m	88	Primary tumor	5.8	pT1b, pN0 (0/11), cM0, L0, V0, Pn0, R0	G2	No information
9	m	69	Primary tumor	7.5	pT3a, pN1 (3/11), cM1 (PUL), L0, V0, Pn0, R0	G2	Stable disease
10	m	79	Primary tumor	5.3	pT3a, pNx, cM0, L0, V0, Pn0, R0	G2	No progress
11	w	55	Primary tumor	8	pT2a, pNx, pM1 (OSS), L0, V1, Pn0, R0	G3	Progressive disease
12	m	63	Primary tumor	2.6	pT1a, pNX, cM0, L0, V0, Pn0, R0	G2	No progress

**Table 2:** PD-L1 status and immune cell densities and proliferation rates at baseline.

**Tumor Number**	**PD-L1 TPS**	**T-Cells/mm^2^**	**Ki67 + T-Cells/mm^2^**	**CTL/mm^2^**	**Ki67 + CTL/mm^2^**	**PD1+ IC/mm^2^**	**Ki67 + PD1+ IC/mm^2^**
1	0	1399.0	41.6	187.2	10.3	109.8	2.2
2	0	453.0	22.5	249.4	27.5	252.0	33.1
3	0	741.2	67.8	235.4	59.1	364.2	91.4
4	0	129.8	4.8	96.1	9.7	39.2	2.6
5	0	4696.5	218.3	592.3	122.6	483.0	158.8
6	0	172.9	9.3	113.7	10.3	129.5	10.9
7	0	273.0	9.3	71.5	3.5	85.2	5.2
8	0	66.0	3.4	46.0	1.8	21.3	1.2
9	0	962.1	103.3	472.2	37.5	812.8	64.4
10	0	1994.3	714.1	1785.0	388.9	1580.6	452.6
11	0	543.4	5.7	352.1	20.3	256.4	21.2
12	0	466.7	5.8	300.9	19.4	564.9	65.3
**Statistics**		**T-cells/mm^2^**	**Ki67 + T-cells/mm^2^**	**CTL/mm^2^**	**Ki67 + CTL/mm^2^**	**PD1+ IC/mm^2^**	**Ki67 + PD1+ IC/mm^2^**
Mean		991.5	100.5	375.2	59.2	391.6	75.7
STD		1297.3	203.2	473.6	109.1	444.3	127.8
Min		66.0	3.4	46.0	1.8	21.3	1.2
Max		4696.5	714.1	1785.0	388.9	1580.6	452.6
Median		505.0	15.9	242.4	19.8	254.2	27.1

Abbreviations: PD-L1: programmed death receptor ligand 1; TPS: tumor proportion score; CTL: CD8+ cytotoxic lymphocytes; PD1+ IC: programmed death receptor 1 expressing immune cells; STD: standard deviation; min: minimum; max: maximum.

**Table 3:** Nivolumab-dependent reaction patterns of tumor infiltrating immune cell densities and corresponding proliferation rates.

Tumor Number	CD3	CD3-Ki67	CD8	CD8-Ki67	PD1	PD1-Ki67
1	~	~	~	~	-	~
2	-	-	~	~	-	-
3	+	+	+	+	-	-
4	+	+	~	~	-	-
5	-	-	-	-	-	-
6	~	~	~	~	-	-
7	~	~	~	~	-	-
8	~	~	-	+	-	NA
9	~	~	-	-	-	~
10	-	-	~	~	-	~
11	+	-	+	+	-	-
12	-	-	~	-	-	-

Abbreviations: CD3: T-cells; CD3-Ki67: proliferating T-cells; CD8: cytotoxic T-cells; CD8-Ki67: proliferating cytotoxic T-cells; PD1: programmed death receptor expressing immune cells; PD1-Ki67: proliferating programmed death receptor expressing immune cells; + nivolumab-dependent increase; - nivolumab-dependent decrease; ~ no nivolumab-dependent change; NA: data not available.

## Data Availability

The data presented in this study are available in this article and Supplementary Materials.

## Supplementary Materials

The following are available online at https://www.mdpi.com/article/10.3390/cancers13184511/s1. Figure S1: Spatial analysis of tumor infiltrating T-cells; Figure S2: Post hoc immunostaining of clear cell renal cell carcinoma (ccRCC); Figure S3: Nivolumab-dependent changes of overall proliferation and tumor necrosis; Figure S4: Nivolumab-dependent changes of overall proliferation, tumor necrosis and tumor infiltrating immune cell densities and proliferation rates; Figure S5: Nivolumab-dependent spatial distribution of tumor infiltrating PD1+ immune cells and T-cells.
