# Supplementary material for: Nivolumab Reduces PD1 Expression and Alters Density and Proliferation of Tumor Infiltrating Immune Cells in a Tissue Slice Culture Model of Renal Cell Carcinoma
Source: Cancers (Basel). 2021 Sep 8;13(18):4511. doi: 10.3390/cancers13184511 (PMC8471479; doi:10.3390/cancers13184511)
Supplement: Supplementary file 1 [file cancers-13-04511-s001.zip › cancers-1371247-supplementary.pdf]

Supplementary Material: Nivolumab Reduces PD1 Expression and Alters Density and Proliferation of Tumor Infiltrating Immune Cells in a Tissue Slice Culture Model of Renal Cell Carcinoma

Philipp J. Stenzel, Nina Hörner, Sebastian Foersch, Daniel-Christoph Wagner, Igor Tsaur, Anita Thomas, Axel Haferkamp, Stephan Macher-Goeppinger, Wilfried Roth, Stefan Porubsky and Katrin E. Tagscherer

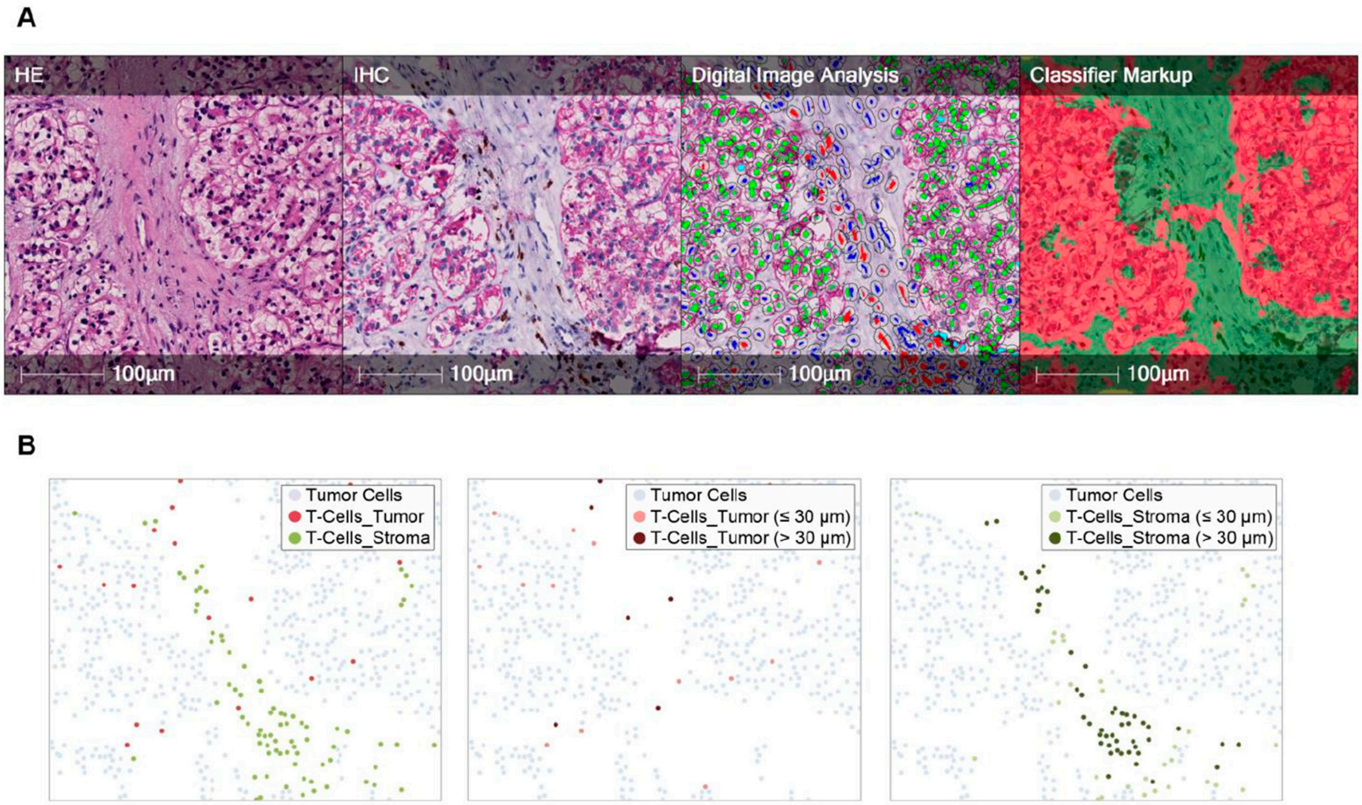

Figure S1. Spatial analysis of tumor infiltrating T-cells. Tumor tissue of clear cell renal cell carcinoma (ccRCC) 10 cultivated for 24 h to 72 h with increasing concentrations of nivolumab. (**A**) Tissue slices were HE-stained (left) and immunohistochemically doublestained for tumor cells (cytokeratin: red) and T-cells (CD3: brown) (middle left). Cells were detected via digital image analysis and classified into tumor cells (green), T-cells (red) and unstained cells (blue) (middle right). The tissue was further classified in tumor parenchyma (red) and tumor-associated stroma (green) (right) using a tissue classifier. (**B**) The distribution of tumor cells (light blue) and T-cells based on the localization in tissue areas classified as tumor parenchyma (T-cells_Tumor: red) or stroma (T-cells_Stroma: green) was plotted in a spatial plot (left). T-cells were further dichotomized into groups with a distance to tumor cells of ≤30 µm and ≥30 µm.

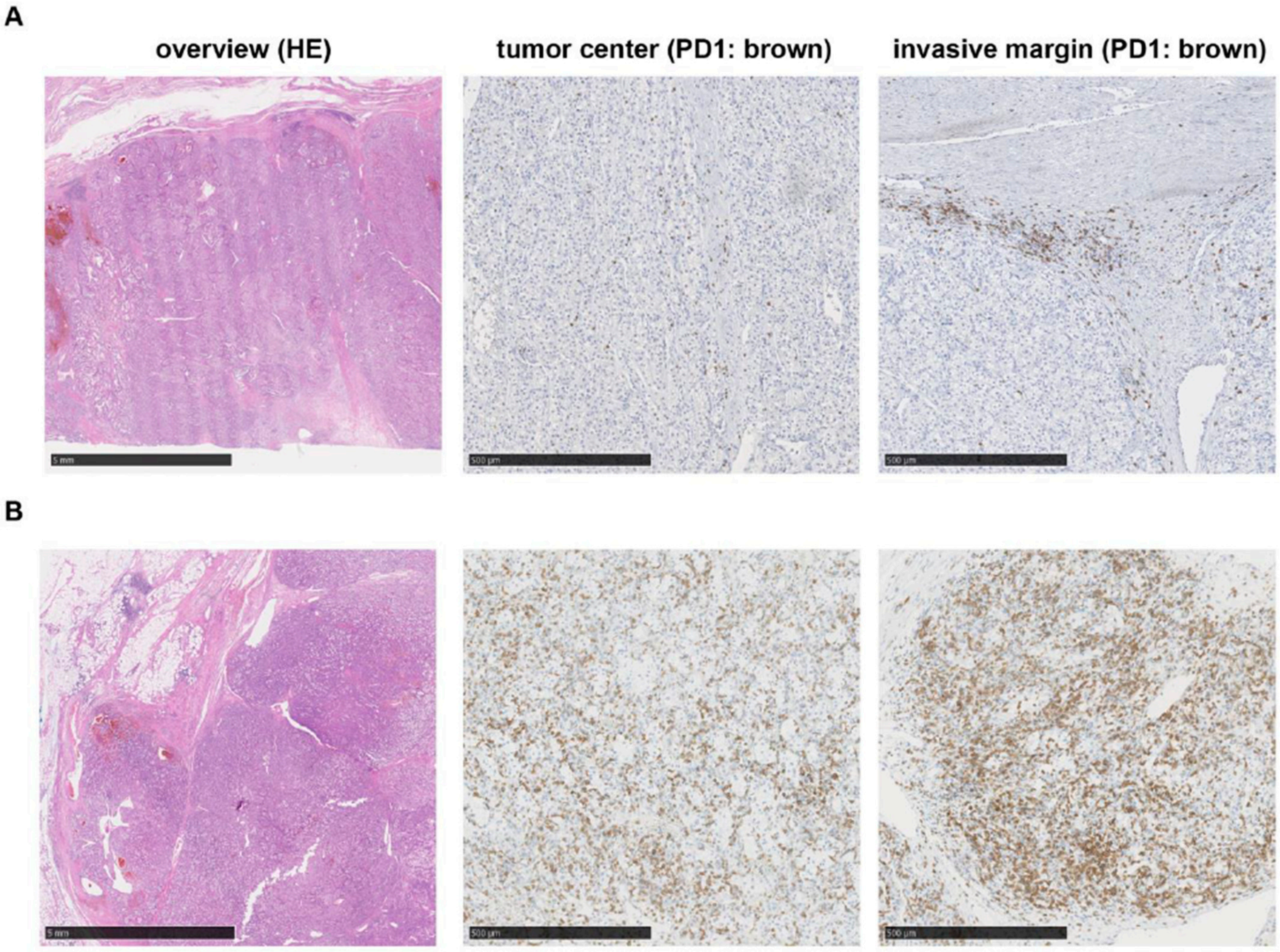

Figure S2. Post-hoc immunostainingof clear cell renal cell carcinoma (ccRCC). Whole slides of ccRCC (left, bar indicates 5 mm) used for tissue slice culture were post-hoc immunostained for programmed death receptor 1 (PD1). Representative images of a tumor with a (**A**) low and (**B**) high infiltrate of PD1+ immune cells in the tumor center and at the invasive margin are shown (bar indicates 500 µm).

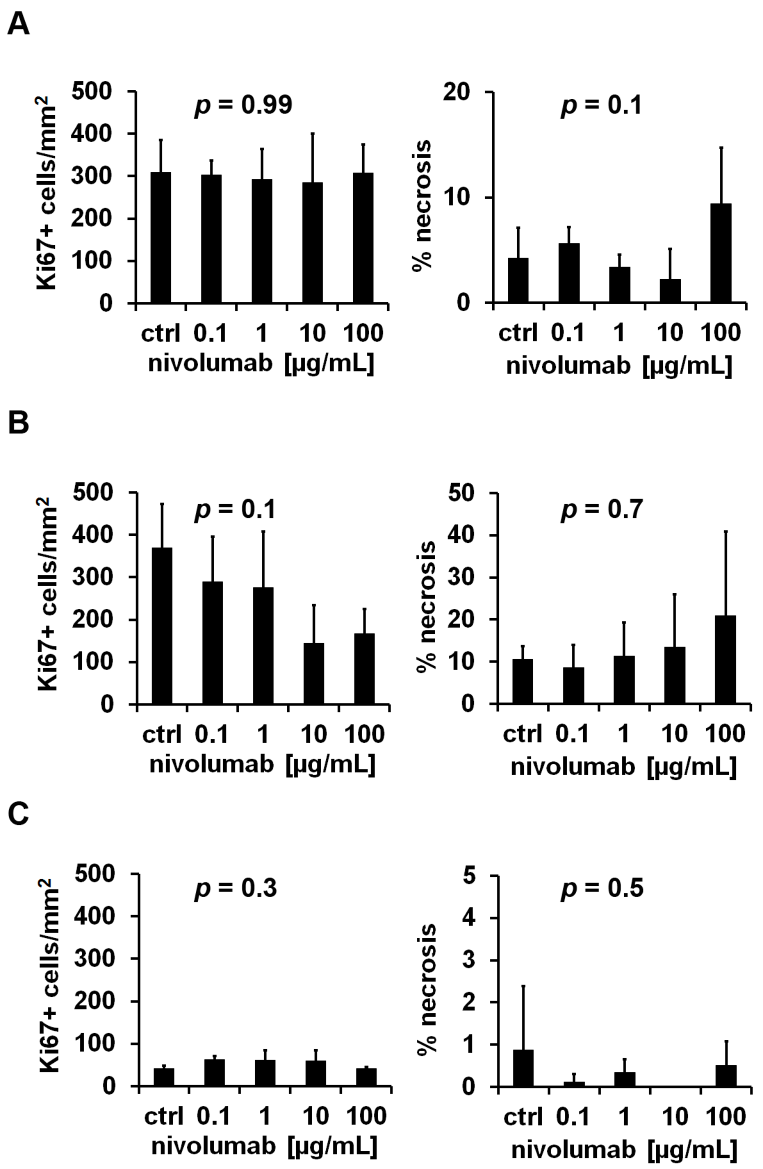

Figure S3. Nivolumab-dependent changes of overall proliferation and tumor necrosis. Tumor tissue of clear cell renal cell carcinoma (ccRCC) was cultivated for 24 h to 72 h with increasing concentrations of nivolumab. Necrotic tumor area and overall proliferation (Ki67) of (**A**) tumor 3, (**B**) Tumor 5 and (**C**) Tumor 7 were quantified by digital image analysis. Data are given as mean ± standard deviation. For statistical analysis the one-way analysis of variance or the Kruskal–Wallis test with appropriate post-hoc tests were used. p-values were corrected with the Bonferroni method.

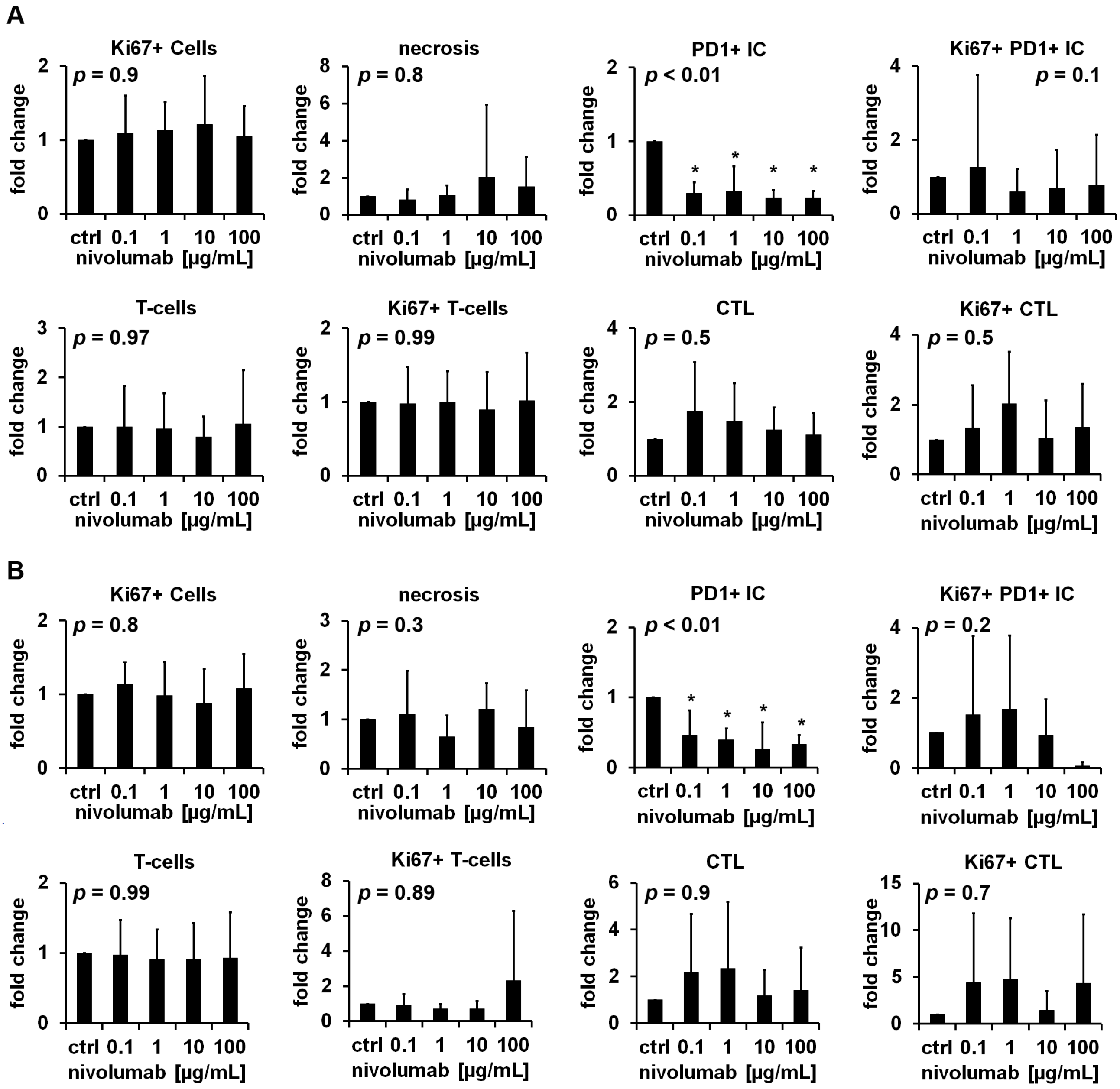

Figure S4. Nivolumab-dependent changes of overall proliferation, tumor necrosis and tumor infiltrating immune cells densities and proliferation rates. Tumor tissue of clear cell renal cell carcinoma (ccRCC) was cultivated for 24 h ($n = 7$) or 72 h ($n = 5$) with increasing concentrations of nivolumab. Tumor necrosis and immunohistochemical stainings for Ki67, Ki67-PD1, Ki67-CD3 and Ki67-CD8 were quantified by digital image analysis. (**A**) Panel A shows the results after treatment for 24 h and (**B**) after 72 h. Data are normalized relative to non-treatment control and given as mean ± standard deviation. For statistical analysis the one-way analysis of variance or the Kruskal–Wallis test with appropriate post-hoc tests were used. *p*-values were corrected with the Bonferroni method. *: $p < 0.05$.

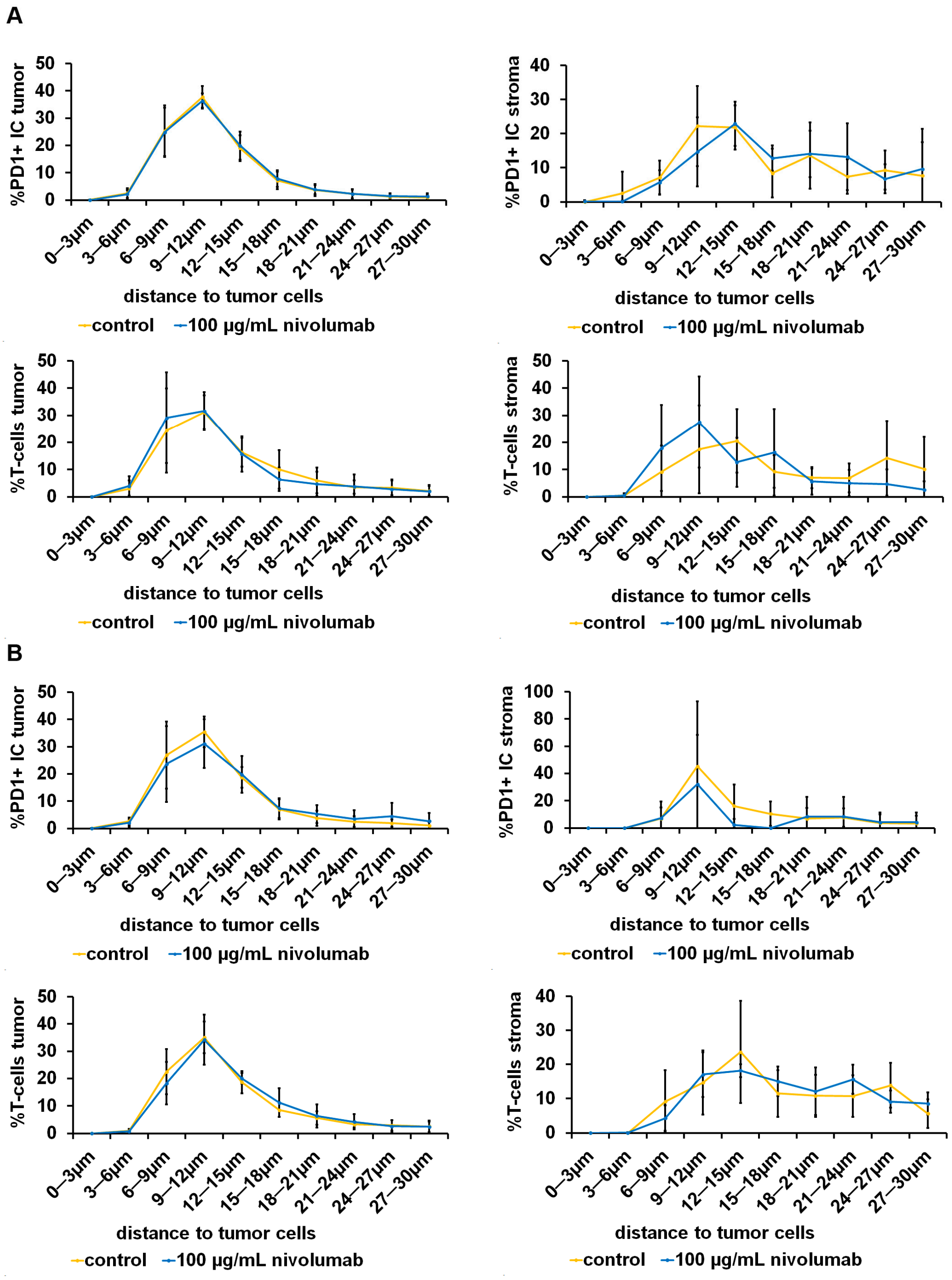

Figure S5. Nivolumab-dependent spatial distribution of tumor infiltrating PD1+ immune cells and T-cells. Tumor tissue of clear cell renal cell carcinoma (ccRCC) cultivated for 24 h to 72 h with nivolumab. Tissue slices were immunohistochemically doublestained for cytokeratin and CD3 or cytokeratin and PD1. The distance between tumor infiltrating or stromal T-cells and PD1+ immune cells to tumor cells was calculated by digital image analysis. (**A**) Distribution after 24 h of nivolumab treatment (n = 7). (**B**) Distribution after 72 h of nivolumab treatment (n = 5). Data are given as mean ± standard deviation.

Publisher's Note: MDPI stays neutral with regard to jurisdictional claims in published maps and institutional affiliations.

© 2021 by the authors. Licensee MDPI, Basel, Switzerland. This article is an open access article distributed under the terms and conditions of the Creative Commons Attribution (CC BY) license (http://creativecommons.org/licenses/by/4.0/).
